# *In Vitro* and *In Vivo* Interactions of TOR Inhibitor AZD8055 and Azoles against Pathogenic Fungi

**DOI:** 10.1128/spectrum.02007-21

**Published:** 2022-01-12

**Authors:** Yi Sun, Lihua Tan, Zhaoqian Yao, Lujuan Gao, Ji Yang, Tongxiang Zeng

**Affiliations:** a Department of Dermatology and Venerology, Jingzhou Hospital, Yangtze University, Jingzhou, Hubei, China; b Department of Dermatology, Xiamen Branch, Zhongshan Hospital Fudan University, Xiamen, China; c Department of Dermatology, Zhongshan Hospital Fudan University, Shanghai, China; University of Debrecen

**Keywords:** TOR inhibitor, fungi, *Aspergillus*, *Candida*, *Exophiala*, *Cryptococcus*, azole, AZD8055, synergy, resistance

## Abstract

In the present study, *in vitro* and *in vivo* interactions of TOR inhibitor AZD8055 and azoles, including itraconazole, voriconazole, posaconazole and fluconazole, against a variety of pathogenic fungi were investigated. A total of 69 isolates were studied via broth microdilution checkerboard technique, including 23 isolates of Aspergillus spp., 20 isolates of *Candida* spp., 9 isolates of Cryptococcus neoformans complex, and 17 isolates of *Exophiala dermatitidis*. The results revealed that AZD8055 individually did not exert any significant antifungal activity. However, synergistic effects between AZD8055 and itraconazole, voriconazole or posaconazole were observed in 23 (33%), 13 (19%) and 57 (83%) isolates, respectively, including azole-resistant A. fumigatus strains and *Candida* spp., potentiating the efficacy of azoles. The combination effect of AZD8055 and fluconazole was investigated against non-auris *Candida* spp. and C. neoformans complex. Synergism between AZD8055 and fluconazole was observed in six strains (60%) of *Candida* spp., resulting in reversion of fluconazole resistance. Synergistic combinations resulted in 4-fold to 256-fold reduction of effective MICs of AZD8055 and azoles. No antagonism was observed. *In vivo* effects of AZD8055-azole combinations were evaluated by survival assay in Galleria mellonella model infected with A. fumigatus strain AF002, *E. dermatitidis* strain BMU00038, C. auris strain 383, C. albicans strain R15, and C. neoformans complex strain Z2. AZD8055 acted synergistically with azoles and significantly increased larvae survival (*P* < 0.05). In summary, the results suggested that AZD8055 combined with azoles may help to enhance the antifungal susceptibilities of azoles against pathogenic fungi and had the potential to overcome azole resistance issues.

**IMPORTANCE** Limited options of antifungals and the emergence of drug resistance in fungal pathogens has been a multifaceted clinical challenge. Combination therapy represents a valuable alternative to antifungal monotherapy. The target of rapamycin (TOR), a conserved serine/threonine kinase from yeast to humans, participates in a signaling pathway that governs cell growth and proliferation in response to nutrient availability, growth factors, and environmental stimuli. AZD8055 is an orally bioavailable, potent, and selective TOR kinase inhibitor that binds to the ATP binding cleft of TOR kinase and inhibits both TORC1 and TORC2. Synergism between AZD8055 and azoles suggested that the concomitant application of AZD8055 and azoles may help to enhance azole therapeutic efficacy and impede azole resistance. TOR inhibitor with fungal specific target is promising to be served as combination regimen with azoles.

## INTRODUCTION

The advancement of chemotherapy and immunomodulation-based therapies have resulted in the rise of the incidence of opportunistic invasive fungal diseases, which are often severe and remain a frequent cause of death in immunosuppressed patients (1). Invasive candidiasis (IC) is the most common health care associated invasive fungal infection (2). Candida albicans remains the most frequent causative agent of IC. However, non-*albicans* species are increasing and associated with less antifungal susceptibilities and outbreaks. Notably, C. auris is an emerging multidrug-resistant pathogen that has caused a certain number of severe infections in recent years and has therefore become a global alarming public health emergency (2). The pathogenic yeast Cryptococcus neoformans and C. gattii comprise the C. neoformans species complex and cause life-threatening cryptococcosis with over 1 million new cases and 600,000 deaths every year (3). Invasive aspergillosis (IA) is the most common mold infection with more than 200,000 cases occur every year and mortality rates of up to 50% even with treatment (1, 4). The most frequent etiologic pathogen of IA is Aspergillus fumigatus (5). Moreover, non-fumigatus Aspergillus spp. with reduced susceptibility to current antifungals constitute a substantial proportion of IA (6). In addition to these common pathogens, dematiaceous fungi *Exophiala dermatitidis*, the leading cause of severe neurotropic phaeohyphomycosis and a common cause of chromoblastomycosis, is also being increasingly recognized and reported (7–9).

Early initiation of appropriate antifungal therapy is crucial to improve patients’ outcome. However, the antifungal choices available are very limited. Only a few classes of antifungals are currently approved for the treatment of invasive mycoses, including azoles, polyenes, echinocandins and flucytosine. In addition, clinical drug resistance has been increasingly reported, which further limits the arsenal of antifungal drugs futile (10). Resistant to either of these classes of antifungals by *Candida* spp., to azoles or amphotericin B by Aspergillus spp. and to fluconazole by C. neoformans complex have been reported worldwide (11). The emergence of multidrug resistance (MDR), which is characterized by simultaneous resistance to at least two distinct classes of antifungal agents, further compromises the treatment options (10). Under such a scenario, combination therapy, which has the potential to potentiate the currently applied antifungals and decrease the probability of development of resistance, represents a valuable and promising alternative option to drug monotherapy.

The target of rapamycin (TOR), a conserved serine/threonine kinase from yeast to humans, participates in a signaling pathway that orchestrates cell growth and proliferation in response to nutrient availability, growth factors, and environmental stimuli (12). It has been demonstrated that the TOR pathway regulates proliferation, translation, transcription, autophagy, ribosome biogenesis, lipid homeostasis, morphogenesis, and cellular aggregation in fungal cells, which have important implications for pathogenicity and virulence (12–15). Therefore, targeting TOR signaling cascade might be an excellent target for the development of broad-spectrum combinational regimen with traditional antifungals. AZD8055 is an orally bioavailable, potent, and selective TOR kinase inhibitor with ∼1,000-fold selectivity against PI3K isoforms or related PIKK family members (16). Hence, it is tempting to speculate that AZD8055 might have antifungal effect or interactions with antifungals against pathogenic fungi. In the present study, the *in vitro* and *in vivo* interactions of AZD8055 with itraconazole (ITC), voriconazole (VRC), posaconazole (POS), or fluconazole (FLC) against pathogenic fungi were investigated.

## RESULTS

### *In vitro* interactions between AZD8055 and azoles against Aspergillus spp.

The MIC of AZD8055 alone against all strains was >64 μg/mL. As shown in Table 1, the MIC ranges of azoles alone against Aspergillus spp. except for azole-resistant strains were 1–4 μg/mL for ITC,0.25–2 μg/mL for VRC, and 0.5–2 μg/mL for POS, respectively. The MIC ranges of azoles were 4->32 μg/mL for ITC, 0.5->32 μg/mL for VRC, and 2–4 μg/mL for POS against azole-resistant A. fumigatus strains.

**TABLE 1:** MICs and FICIs results with the combinations of AZD8055 and azoles against Aspergillus spp

	MIC^*a*^ (μg/mL) for						
	Agent alone				Combination^*b*^		
Strains	AZD8055	ITC	VRC	POS	AZD8055/iTC	AZD8055/vRC	AZD8055/pOS
A. fumigatus							
AF293	>64	1	0.5	1	64/0.5(1, I)	1/0.5(1.008, I)	16/0.25(0.375, S)
AF001	>64	4	1	2	16/1(0.375, S)	4/0.25(0.281, S)	8/0.25(0.188, S)
AF002	>64	4	1	2	16/2(0.625, I)	1/1(1.008, I)	32/0.5(0.5, S)
AF003	>64	4	1	1	32/1(0.5, S)	8/0.5(0.563, I)	16/0.25(0.375, S)
AF004	>64	2	0.25	1	32/0.5(0.5, S)	1/0.25(1.008, I)	16/0.25(0.375, S)
AF005	>64	2	1	1	32/0.5(0.5, S)	16/0.25(0.375, S)	4/0.25(0.281, S)
AF006	>64	2	1	1	64/1(1, I)	1/1(1.008, I)	16/0.25(0.375, S)
AF007	>64	1	0.5	1	32/0.25(0.5, S)	1/0.5(1.008, I)	16/0.25(0.375, S)
AF008	>64	2	0.5	1	16/1(0.625, I)	64/0.25(1, I)	16/0.25(0.375, S)
AF009	>64	2	0.25	0.5	32/0.5(0.5, S)	1/0.25(1.008, I)	16/0.125(0.375, S)
AF010	>64	2	0.25	1	16/1(0.625, I)	1/0.25(1.008, I)	8/0.25(0.313, S)
AF011	>64	1	1	1	16/0.5(0.625, I)	1/1(1.008, I)	16/0.25(0.375, S)
AF012	>64	1	1	0.5	16/0.5(0.625, I)	1/1(1.008, I)	16/0.25(0.625, I)
AF013	>64	1	2	0.5	32/0.5(0.75, I)	1/2(1.008, I)	16/0.25(0.625, I)
R1(TR34/L98H)	>64	>32	4	2	64/32(1, I)	1/4(1.008, I)	32/1(0.75, I)
R2(TR34/L98H)	>64	>32	0.5	2	64/32(1, I)	1/0.5(1.008, I)	16/1(0.625, I)
R3(TR34/L98H)	>64	>32	4	4	64/32(1, I)	1/2(0.508, I)	16/1(0.375, S)
R4(TR46/Y121F/T 289A)	>64	4	>32	4	1/2(0./508, I)	16/4(0.313, S)	16/1(0.375, S)
A. flavus							
AFLA-1	>64	2	1	1	1/2(1.016, I)	1/1(1.008, I)	16/0.25(0.375, S)
AFLA-2	>64	2	2	1	32/1(0.75, I)	1/2(1.008, I)	16/0.25(0.375, S)
AFLA-3	>64	1	0.5	1	16/0.5(0.625,I)	1/0.5(1.008, I)	32/0.25(0.5, S)
A. terreus							
AT-1	>64	2	1	1	1/1(0.508, I)	1/1(1.008, I)	2/0.5(0.516, I)
AT-1	>64	2	1	1	16/1(0.625, I)	1/1(1.008, I)	16/0.25(0.375, S)

a The MIC is the concentration achieving 100% growth inhibition.

b FICI results are shown in parentheses. S, synergy (FICI of ≤ 0.5); I, no interaction (indifference) (0.5<FICI ≤ 4). For FICI calculations, the concentration of 128 μg/mL and 64 μg/mL were used when MICs were >64 μg/mL and >32 μg/mL, respectively.

When AZD8055 was combined with ITC, VRC or POS, synergistic activity was observed in 6 (26%), 3 (13%), 18 (78%) strains of Aspergillus species isolates (Table 1, 2). Notably, the AZD8055-VRC and AZD8055-POS combinations also showed synergy against azole-resistant A. fumigatus strains, resulting in up to 16-fold reduction of the MICs of azoles. The MICs of AZD8055 and ITC against Aspergillus spp. in the synergistic combinations decreased to 16–32 μg/mL and 0.25–1 μg/mL, respectively (Table 1). When AZD8055 was combined with VRC, the effective working ranges of AZD8055 and VRC were 4–16 μg/mL and 0.25–4 μg/mL, respectively (Table 1). In synergistic AZD8055-POS combination, the MIC ranges of AZD8055 and POS decreased to 4–32 μg/mL and 0.125–1 μg/mL, respectively. No antagonism was observed in all combinations.

**TABLE 2:** Summary of drug interaction for the combination of AZD8055 and azoles

Species(n)	n (%) of isolates showing synergism for the combination			
	AZD8055+iTC	AZD8055+vRC	AZD8055+pOS	AZD8055+fLC
Aspergillus spp. (23)	6 (26%)	3 (13%)	18 (78%)	
A. fumigatus (18)	6 (33%)	3 (17%)	14 (78%)	
A. flavus (3)	0	0	3 (100%)	
A. terreus (2)	0	0	1 (50%)	
*E. dermatitidis* (17)	2 (12%)	1 (6%)	16 (94%)	
*Candida* spp. (20)	13 (65%)	8 (40%)	18 (90%)	
C. albicans (6)	3 (50%)	2 (23%)	5 (83%)	3 (50%)
C. auris (10)	9 (90%)	6 (60%)	9 (90%)	
Other Candida species (4)	1 (25%)	0	4 (100%)	3 (75%)
C. neoformans complex (9)	2 (22%)	1 (11%)	5 (56%)	0
Total (69)	23 (33%)	13 (19%)	57 (83%)	

### *In vitro* interactions between AZD8055 and azoles against *E.dermatitidis*.

The individual MIC ranges of tested agents against *E. dermatitidis* were >64 μg/mL,1–2 μg/mL,0.06–1 μg/mL, and 0.5–1 μg/mL for AZD8055, ITC, VRC and POS, respectively (Table 3). When AZD8055 was combined with ITC, VRC or POS, synergy was observed in 2 (12%),1 (6%) and 16 (94%) strains of *E. dermatitidis* isolates, respectively (Table 2, 3). The MICs of AZD8055, ITC, VRC and POS in the synergistic combinations decreased to 2–32 μg/mL,0.25–0.5 μg/mL, 0.125 μg/mL, and 0.125–0.25 μg/mL, respectively. No antagonism was observed in all combinations.

**TABLE 3:** MICs and FICIs results with the combinations of AZD8055 and azoles against *E. dermatitidis*

Strains	MIC^*a*^ (μg/mL) for						
	Agent alone				Combination^*b*^		
	AZD8055	ITC	VRC	POS	AZD8055/iTC	AZD8055/vRC	AZD8055/pOS
BMU00028	>64	1	0.125	0.5	4/0.5(0.531, I)	1/0.125(1.008, I)	32/0.125(0.5, S)
BMU00029	>64	2	1	1	16/1(0.625, I)	1/1(1.008, I)	4/0.25(0.281, S)
BMU00030	>64	1	0.25	1	64/0.5(1, I)	1/0.25(1.008, I)	32/0.25(0.5, S)
BMU00031	>64	2	0.5	1	32/1(0.75, I)	1/0.5(1.008, I)	8/0.25(0.313, S)
BMU00034	>64	2	0.5	0.5	1/2(1.008, I)	1/0.5(1.008, I)	4/0.25(0.531, I)
BMU00035	>64	1	1	1	16/1(1.008, I)	1/0.125(0.133, I)	4/0.25(0.281, S)
BMU00036	>64	1	0.125	1	16/0.5(0.625, I)	1/0.125(1.008, I)	32/0.25(0.5, S)
BMU00037	>64	1	0.25	1	64/0.5(1, I)	1/0.25(1,0.008, I)	16/0.25(0.375, S)
BMU00038	>64	2	0.5	1	32/0.5(0.5, S)	32/0.125(0.5, S)	32/0.25(0.5, S)
BMU00039	>64	1	0.25	1	64/0.5(1, I)	1/0.125(0.508, I)	32/0.25(0.5, S)
BMU00041	>64	1	0.125	0.5	8/0.5(0.563, I)	1/0.125(1.008, I)	16/0.125(0.375, S)
109140	>64	2	0.125	1	64/1(1, I)	1/0.125(1.008, I)	8/0.25(0.313, S)
109144	>64	2	0.125	1	8/1(0.563, I)	1/0.25(2.008, I)	8/0.25(0.313, S)
109145	>64	2	0.125	1	8/1(0.563, I)	1/0.25(2.008, I)	16/0.125(0.25, S)
109148	>64	2	0.125	1	8/1(0.563, I)	1/0.125(1.008, I)	8/0.125(0.188, S)
109149	>64	2	0.125	0.5	8/1(0.563, I)	1/0.125(1.008, I)	8/0.125(0.313, S)
109152	>64	1	0.06	0.5	32/0.25(0.5, S)	1/0.06(1.008, I)	2/0.125(0.266, S)

a The MIC is the concentration achieving 100% growth inhibition.

b FICI results are shown in parentheses. S, synergy (FICI of ≤ 0.5); I, no interaction (indifference) (0.5<FICI ≤ 4). For FICI calculations, the concentration of 128 μg/mL were used when MICs were >64 μg/mL.

### *In vitro* interactions between AZD8055 and azoles against *Candida* spp.

The individual MIC ranges of AZD8055, ITC, VRC and POS were >64 μg/mL,0.25–16 μg/mL,0.25–16 μg/mL and 0.06–4 μg/mL, respectively (Table 4). The MIC range of FLC against non-auris *Candida* spp. were 2->64 μg/mL. When AZD8055 was combined with ITC, VRC or POS, synergistic activity was observed in 4 (40%), 2 (20%), and 9 (90%) strains of non-auris *Candida* spp. and 9 (90%), 6 (60%), and 9 (90%) strains of C. auris, respectively (Table 2, 4). The MICs of AZD8055 and ITC in synergistic combination decreased to 4–32 μg/mL and 0.125–2 μg/mL, respectively (Table 4). When AZD8055 was combined with VRC, the effective MIC ranges of AZD8055 and VRC decreased to 4–32 μg/mL and 0.25–4 μg/mL, respectively. When AZD8055 was combined with POS, the effective working ranges of AZD8055 and POS were 2–32 μg/mL and 0.06–1 μg/mL, respectively (Table 4). Synergism between AZD8055 and FLC was observed in 6 strains of non-auris *Candida* spp. The effective MIC ranges of AZD8055 and FLC against non-auris *Candida* spp. were 1–2 μg/mL and 0.5–4 μg/mL, respectively. It is worth noting that synergistic effect of AZD8055 and azoles resulted in up to 256-fold reduction in the MICs of azoles. No antagonism was observed in all combinations.

**TABLE 4:** MICs and FICIs results with the combinations of AZD8055 and azoles against *Candida* spp

Strains	MIC^*a*^ (μg/mL) for								
	Agent alone					Combination^*b*^			
	AZD8055	ITC	VRC	POS	FLC	AZD8055/iTC	AZD8055/vRC	AZD8055/pOS	AZD8055/fLC
C. auris									
381	>64	1	0.125	0.25		2/0.5(0.516,I)	1/0.125(1.008, I)	16/0.06(0.365, S)	
382	>64	1	4	1		32/0.25(0.5, S)	32/2(0.75, I)	2/0.06(0.076, S)	
383	>64	1	4	1		32/0.125(0.375, S)	16/0.5(0.25, S)	4/0.25(0.281, S)	
384	>64	1	4	0.25		8/0.25(0.313, S)	32/2(0.75, I)	16/0.125(0.625, I)	
385	>64	2	8	2		4/0.5(0.281, S)	32/2(0.5, S)	4/0.25(0.156, S)	
386	>64	2	16	1		32/0.5(0.5, S)	32/4(0.5, S)	4/0.25(0.281, S)	
387	>64	2	16	1		16/0.5(0.375, S)	4/0.25(0.063, S)	4/0.25(0.281, S)	
388	>64	2	2	0.5		16/0.5(0.375, S)	32/0.25(0.375, S)	16/0.125(0.375, S)	
389	>64	1	4	0.5		32/0.25(0.5, S)	1/4(1.008, I)	32/0.06(0.37, S)	
390	>64	1	1	0.5		32/0.125(0.375, S)	16/0.25(0.375, S)	4/0.125(0.281, S)	
C. albicans									
R2	>64	2	4	0.06	2	1/1(0.508, I)	1/2(0.508, I)	1/0.06(1.008, I)	2/0.5(0.266, S)
R9	>64	16	8	4	2	8/2(0.188, S)	4/2(0.281,S)	4/1(0.281, S)	1/2(1.008, I)
R14	>64	4	4	1	32	2/1(0.266, S)	1/4(1.008, I)	2/0.25(0.266, S)	1/4(0.133, S)^*c*^
R15	>64	4	8	0.5	16	16/1(0.375, S)	16/2(0.375, S)	8/0.125(0.313, S)	1/4(0.258, S)^*c*^
R65	>64	0.5	0.25	1	8	1/0.5(1.008, I)	1/0.25(1.008, I)	8/0.125(0.188, S)	1/4(0.508, I)
ATCC64550	>64	2	2	1	16	16/1(0.625, I)	32/1(0.75, I)	4/0.25(0.281, S)	16/8(−0.508, I)
C. tropicalis									
BMU05150	>64	0.5	1	0.5	4	32/0.25(0.75, I)	1/1(1.008, I)	8/0.125(0.313, S)	1/0.5(0.133, S)^*d*^
C. krusei									
ATCC00279	>64	1	4	0.5	>64	32/0.25(0.5, S)	16/2(0.625, I)	16/0.125(0.375, S)	1/0.5(0.012, S)
C. parapsilosis									
ATCC22019	>64	0.25	0.25	0.5	0.5	16/0.125(0.625, I)	1/0.25(1.008, I)	4/0.125(0.281, S)	1/0.5(1.008, I)
C. glabrata									
BMU05448	>64	4	2	1	16	32/2(0.75, I)	16/1(0.625, I)	16/0.125(0.25, S)	1/0.5(0.039, S)

a The MIC is the concentration achieving 50% growth inhibition.

b FICI results are shown in parentheses. S, synergy (FICI of ≤ 0.5); I, no interaction (indifference) (0.5<FICI ≤ 4). For FICI calculations, the concentration of 128 μg/mL were used when MICs were >64 μg/mL.

c Category change from resistance to susceptible dose dependent (SDD).

d Category change from SDD to susceptible. Susceptible/SDD/resistant is defined as an MIC ≤2/4/≥8 mg/liter of fluconazole for C. albicans, C. tropicalis and C. parapsilosis, and an MIC of 32 and ≥64 mg/liter of fluconazole is defined as SDD and resistant for C. glabrata, respectively (38). Susceptible/SDD/resistant is defined as an MIC ≤0.125/0.25 – 0.5/≥1 mg/liter of voriconazole for C. albicans, C. tropicalis and C. parapsilosis, and an MIC ≤0.5/1/≥2 mg/liter of voriconazole for C. krusei (38). Susceptible/SDD/resistant is defined as an MIC ≤0.125/0.25 – 0.5/≥1 mg/liter of itraconazole for C. albicans (38). Category change was analyzed for those species with CLSI clinical breakpoint.

### *In vitro* interactions between AZD8055 and azoles against C. neoformans complex.

The individual MIC ranges for AZD8055, ITC, VRC, POS and FLC against C. neoformans complex were >64 μg/mL,0.25–2 μg/mL,0.03–1 μg/mL,0.06–1 μg/mL, and 4–16 μg/mL, respectively (Table 5). When AZD8055 was combined with ITC, VRC or POS, synergy was observed in 2 (22%), 1 (11%) and 5 (56%) strains, respectively (Table 2, 5). The MICs of AZD8055, ITC, VRC, and POS in synergistic combination decreased to 2–32 μg/mL,0.06–0.125 μg/mL, 0.25 μg/mL, and 0.03–0.25 μg/mL, respectively. No antagonism was observed in all combinations.

**TABLE 5:** MICs and FICIs results with the combinations of AZD8055 and azoles against C. neoformans complex

Strains	MIC^*a*^ (μg/mL) for								
	Agent alone					Combination^*b*^			
	AZD8055	ITC	VRC	POS	FLC	AZD8055/iTC	AZD8055/vRC	AZD8055/pOS	AZD8055/fLC
Z1	>64	0.25	0.03	0.5	8	32/0.125(0.75, I)	1/0.03(1.008, I)	2/0.125(0.266, S)	1/8(1.008, I)
Z2	>64	0.25	0.06	0.25	8	2/0.06(0.256, S)	16/0.03(0.625, I)	16/0.06(0.365, S)	1/8(1.008, I)
Z3	>64	0.25	0.125	0.25	16	32/0.125(0.75, I)	64/0.06(0.98, I)	8/0.03(0.183, S)	1/16(1.008, I)
G5	>64	2	0.25	1	16	32/1(0.75, I)	1/0.25(1.008, I)	32/0.25(0.5, S)	1/16(1.008, I)
G6	>64	0.25	0.03	0.06	4	1/0.125(0.508, I)	1/0.03(1.008, I)	1/0.06(1.008,I)	1/4(1.008, I)
G7	>64	2	1	1	8	16/1(0.625, I)	1/1(1.008, I)	8/0.5(0.563, I)	1/4(0.508, I)
G8	>64	0.5	0.06	0.5	8	2/0.125(0.266, S)	1/0.06(1.008, I)	4/0.06(0.151, S)	1/4(0.508, I)
G9	>64	0.5	0.06	0.5	16	2/0.25(0.516, I)	1/0.06(1.008, I)	2/0.25(0.516, I)	1/8(0.508, I)
G10	>64	2	1	1	8	32/1(0.75, I)	32/0.25(0.5, S)	16/0.5(0.625, I)	1/8(1.008, I)

a The MIC is the concentration achieving 50% growth inhibition.

b FICI results are shown in parentheses. S, synergy (FICI of ≤ 0.5); I, no interaction (indifference) (0.5<FICI ≤ 4). For FICI calculations, the concentration of 128 μg/mL were used when MICs were >64 μg/mL.

### *In vivo* effects of AZD8055 alone and combined with azoles against *A. fumigatus*.

**FIG 1:**
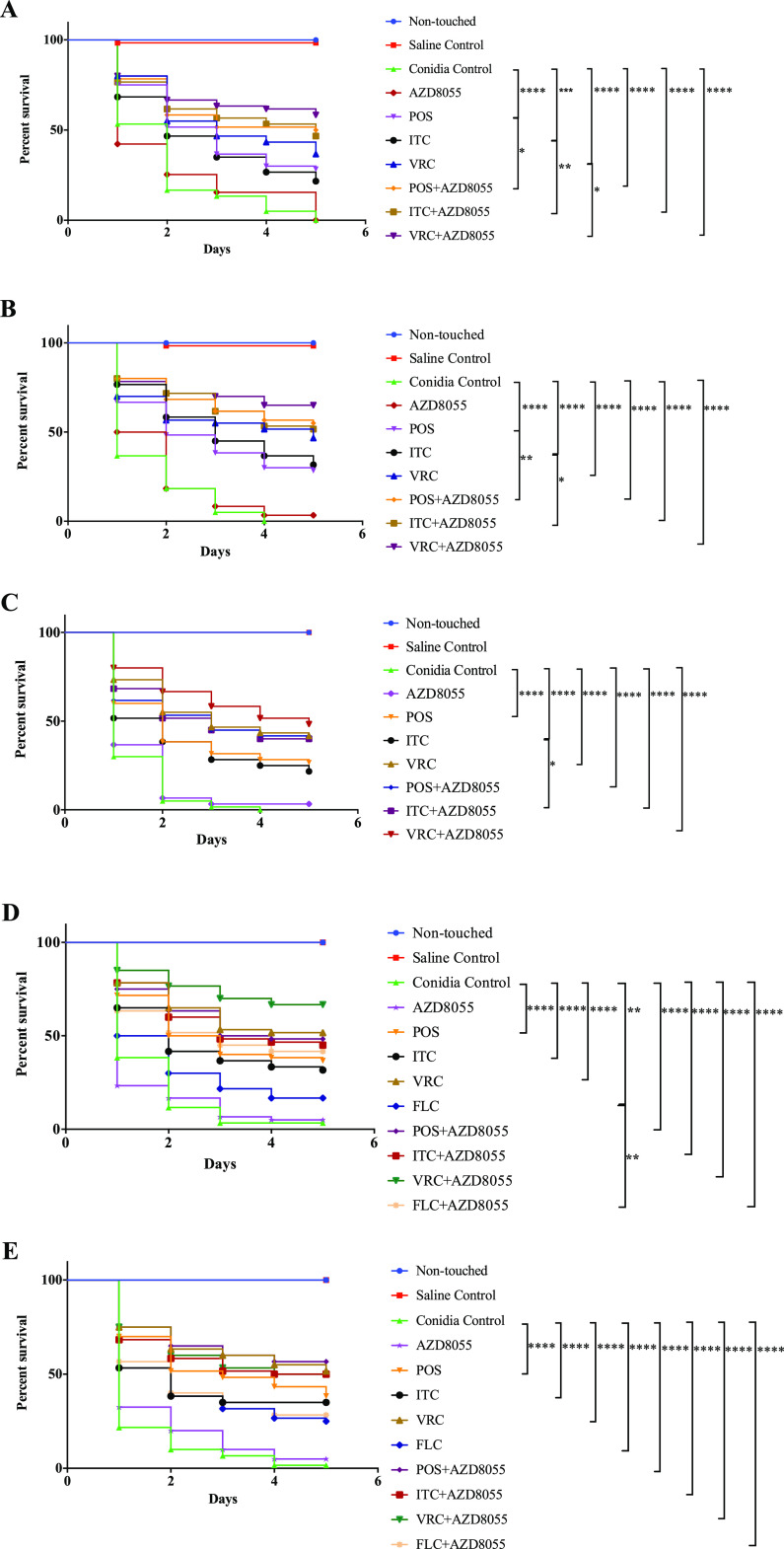
Survival curve of G. mellonella infected with pathogenic fungi. (A) A. fumigatus AF002, (B) *E. dermatitis* BMU00038, (C) C. auris 383, (D) C. albicans R15, (E) C. neoformans complex Z2. ****, *P* < 0.0001; ***, *P* < 0.001; **, *P* < 0.01; *, *P* < 0.05.

The survival rates of larvae infected with A. fumigatus in groups treated with POS, ITC, VRC, AZD8055, AZD8055 with POS, AZD8055 with ITC, and AZD8055 with VRC were 28.3%, 21.7%, 36.7%, 0%, 50%, 46.7%, and 58.3%, respectively. Treatment with azoles alone and combined with AZD8055 all significantly (*P* < 0.001) prolonged the survival of larvae (Fig. 1A). The combinations of AZD8055 with azoles acted synergistically against A. fumigatus infection, compared to azoles alone, respectively (*P* < 0.05).

### *In vivo* effects of AZD8055 alone and combined with azoles against *E. dermatitidis*.

The survival rates of larvae treated with POS, ITC, VRC, AZD8055, AZD8055 with POS, AZD8055 with ITC, and AZD8055 with VRC were 28.3%, 31.7%, 46.7%, 3.3%, 55%, 51.7% and 65%, respectively. Treatment with azoles alone or combined with AZD8055 all significantly (*P* < 0.0001) prolonged the survival of larvae infected with *E. dermatitidis* (Fig. 1B). Treatment with AZD8055 alone showed no effect on *E. dermatitidis* infection. However, in groups that received AZD8055 combined with POS or ITC, the survival of larvae were significantly (*P* < 0.05) prolonged compared to groups that received POS or VRC only, respectively (Fig. 1B).

### *In vivo* effects of AZD8055 alone and combined with azoles against C. auris.

The survival rates of larvae in groups treated with POS, ITC, VRC, AZD8055, AZD8055 with POS, AZD8055 with ITC, and AZD8055 with VRC were 26.7%, 21.7%, 41.7%, 3.3%, 41.7%, 40% and 48.3%, respectively. Treatment with AZD8055 alone showed no effect on C. auris infection. Treatment with azoles alone or combined with AZD8055 all significantly (*P* < 0.0001) prolonged the survival of larvae infected with C. auris (Fig. 1C). In addition, the combination of ITC with AZD8055 significantly (*P* < 0.05) prolonged the survival of larvae compared to the group that received ITC only (Fig. 1C).

### *In vivo* effects of AZD8055 alone and combined with azoles against C. albicans.

The survival rates of larvae in groups treated with POS, ITC, VRC, FCL, AZD8055, AZD8055 with POS, AZD8055 with ITC, AZD8055 with VRC, and AZD8055 with FLC was 36.7%, 31.7%, 51.7%, 16.7%, 5%, 48.3%, 45%, 66.7% and 41.7%, respectively. Treatment with AZD8055 alone showed no effect on C. albicans infection. Treatment with azoles alone or combined with AZD8055 all significantly (*P* < 0.01 for FLC alone group and *P* < 0.0001 for other groups) prolonged the survival of larvae infected with C. albicans (Fig. 1D). In addition, the combination of FLC with AZD8055 significantly (*P* < 0.01) prolonged the survival of larvae compared to the group that received FLC only (Fig. 1D).

### *In vivo* effects of AZD8055 alone and combined with azoles against C. neoformans complex.

The survival rates of larvae in groups treated with POS, ITC, VRC, FCL, AZD8055, AZD8055 with POS, AZD8055 with ITC, AZD8055 with VRC, and AZD8055 with FLC was 38.3%, 35%, 51.7%, 25%, 5%, 56.7%, 50%, 50%, and 28.3%, respectively. Treatment with AZD8055 alone showed no effect on C. neoformans complex infection. Treatment with azoles alone or combined with AZD8055 all significantly (*P* < 0.0001) prolonged the survival of larvae infected with C. neoformans complex (Fig. 1E). However, there was no significant difference in the survival rate of larvae between azoles alone groups and combination groups (Fig. 1E).

## DISCUSSION

The globally conserved TOR signaling cascade has been widely studied since its discovery and has been recognized as a central controller of cell growth and proliferation in eukaryotes (12). TOR, the first defined member of the PI3K-like kinase (PIKK) family, physically large serine/threonine kinases and the central element of TOR signaling pathway, were first identified in Saccharomyces cerevisiae as the target of the antifungal and immunosuppressive agent rapamycin (17, 18). It functions in distinct multiprotein complexes named TORC1 and TORC2 (19). Investigations have shown that TOR signaling pathway plays important roles in the pathogenesis of fungi (14, 20). TOR regulates the expression of genes associated with morphogenesis, cellular adhesion and aggregation, which have implications for the virulence of C. albicans (13, 20). It has also been demonstrated in C. neoformans that TOR pathway plays pleiotropic roles in growth, thermotolerance and DNA damage response (21).

Previously, rapamycin, the classical allosteric inhibitor of TOR, was demonstrated *in vitro* to exhibit potent antifungal efficacy against a variety of species, including *Candida* spp., Aspergillus spp., Cryptococcus spp., Fusarium spp., *Penicillium* spp., and dermtophytes (22). In addition, synergism between rapamycin and amphotericin B or azoles against *Mucorales* was reported (23). However, information regarding the combination effects of new-generation TOR inhibitor and traditional antifungals against yeast and antifungal resistant fungi remains elusive. It is proverbial that rapamycin is an allosteric inhibitor of TORC1 and does not affect TORC2. In contrast, AZD8055 binds to the ATP binding cleft of TOR kinase and inhibits both TORC1 and TORC2. Previous studies have shown superior pharmacokinetic, activity and excellent selectivity profiles of AZD8055 (16, 24).

In the present study, we investigated the *in vitro* and *in vivo* interactions of AZD8055 with azoles against a variety of pathogenic fungi, including azole-resistant strains of A. fumigatus and *Candida* spp. A total of 69 strains were studied *in vitro*. Although AZD8055 alone did not exert any significant antifungal activity, synergistic effects between AZD8055 and ITC, VRC or POS were observed in 23 (33%), 13 (19%) and 57 (83%) strains, respectively (Table 2). Among different azoles, synergy was most often observed in the combination between AZD8055 and POS. The interaction between AZD8055 and FLC were only investigated against non-auris *candida* spp. and C. neoformans complex. Synergism between AZD8055 and FLC was observed in 6 strains (60%) of *Candida* spp. It is worth noting that AZD8055-VRC/POS combination exerted synergism against azole-resistant A. fumigatus strains harboring the association of a tandem repeat sequence and punctual mutation of the Cyp51A gene (TR34/L98H and TR46/Y121F/T289A) and resulted in up to 16-fold reduction in MICs of azoles. In addition, synergistic effects were frequently observed against multidrug-resistant C. auris with up to 64-fold reduction of MICs of azoles. As for C. albicans and C. tropicalis, the combination of AZD8055 and FLC have resulted in category change of FLC susceptibilities (from resistant to susceptible dose dependent [SDD], and SDD to susceptible, respectively) (Table 4).

The *in vitro* data were further confirmed *in vivo* since the combination of azoles and AZD8055 all showed significant (*P* < 0.0001) improvement in larvae survival compared to control groups. No significant increase in larvae survival due to AZD8055 application alone could be detected. All azoles applied alone also significantly (*P* < 0.001) increased larvae survival in all tested isolate. In addition, the application with AZD8055 and ITC resulted in significant increase of survival compared to ITC application alone against A. fumigatus, *E. dermatitidis* and C. auris infection (*P* < 0.05). The AZD8055-POS combination significantly improved survival of larvae infected with A. fumigatus and *E. dermatitidis*, compared to POS alone groups (*P* < 0.05). The AZD8055-VRC significantly improved survival of larvae infected with A. fumigatus, compared to VRC alone groups (*P* < 0.05). The combination of AZD8055 and FLC significantly enhanced the antifungal effect against larvae infected with C. albicans, compared to FLC alone (*P* < 0.05). As for C. neoformans complex infection, there is no significant difference in survival rates between combination groups and azole alone groups. However, in accordance with *in vitro* susceptibilities that showed synergy of AZD8055-POS/ITC combinations and indifference of AZD8055-VRC/FLC combinations against C. neoformans complex, higher survival of larvae in groups treated with AZD8055-POS/ITC was observed in comparison to groups treated with POS/ITC alone, while comparable survival rates were observed among AZD8055-VRC/FLC and VRC/FLC groups.

*Candida*, Aspergillus, and Cryptococcus species compromise the majority of fungal infections. It is worth mentioning that other fungal species, including dematiaceous fungi, *Zygomycete*, Fusarium spp. are also assuming clinical significance, being responsible for fatal diseases. Azoles are the most widely deployed antifungals for the therapy of fungal infections in clinical practice. However, the emergence of azole resistance and azole-inactive pathogenic fungi result in therapeutic failures and continue to be a growing problem in the medical community (11). Therefore, it is encouraging to find that the combinations of AZD8055 with azoles exerted synergistic effects and potentiated the effect of azoles *in vitro* and *in vivo*, resulting in reversion of azole resistance.

Hsp90, a molecular chaperone that stabilizes the calcineurin protein, coordinates cellular circuitry critical for responses to antifungal-induced stress and plays an essential role in antifungal drug resistance (25, 26). Inactivation of Hsp90 is essential for cells to survive in the presence of azoles, converting azoles from fungistatic to fungicidal (25, 26). Previous study has shown that inhibition of Tor1 leads to inhibition of Hsp90 activity, resulting in hypersensitivity to azoles in S. cerevisiae and C. albicans (27). In the contrary, TOR signaling hyperactivation led to azole resistance by stabilizing calcineurin via activation of Hsp90 (28). Therefore, we suspected that inhibition of TOR signaling by AZD8055 potentiated azole activity and rendered azole-resistant fungi responsive to azoles via compromising Hsp90 function. However, further investigations are needed to address critical mechanistic questions.

In conclusion, the study extended previous findings in the combination effects between TOR inhibitors and azoles. The results highlighted that the concomitant application of AZD8055 and azoles may help to enhance azole therapeutic efficacy and impede azole resistance, suggesting that TOR inhibitor with fungal specific target is promising to be served as combination regimen with azoles. On the other hand, AZD8055 was originally developed as therapeutic agent for tumor treatment. Since patients undergoing anti-tumor treatment are more predisposed to invasive mycosis, this *in vitro* interaction profile might help clinicians chose more proper antifungal treatments with AZD8055. However, the limitation of the present study is the sample size of some species studied. More species and isolates involving variant phenotypes and genotypes are warranted to investigate the comprehensive profile of the effects of AZD8055 alone and in combination with azole and to evaluate the potential for concomitant use of these agents in humans.

## MATERIALS AND METHODS

### Fungal strains.

A total of 69 strains were studied, including of 23 strains of Aspergillus spp., 20 strains of *Candida* spp., 9 strains of C. neoformans complex, and 17 strains of *E. dermatitidis.* C. parapsilosis (ATCC 22019) and A. flavus (ATCC 204304) were included to ensure quality control. All fungal strains were identified by microscopic morphology and by molecular sequencing of the internal transcribed spacer (ITS) ribosomal DNA (rDNA) (29). For identification of Aspergillus spp., additional molecular sequence of β-tubulin and calmodulin were required (30, 31).

### Antifungals and chemical agents.

All tested agents including AZD8055, ITC, VRC, POS, and FLC were purchased in powder form from Selleck Chemicals, Houston, TX, USA and diluted in dimethyl sulfoxide as stock solutions (3200 μg/mL).

### *In vitro* interactions of AZD8055 and azoles against pathogenic fungi.

Susceptibility testing was performed according to the broth microdilution chequerboard procedure based on the CLSI M27-A3 (32) and M38-A2 (33) standard and previously published protocols (34). For yeast, conidia harvested from cultures grown for 2 days on Sabouraud dextrose agar (SDA) were suspended in sterile distilled water containing 0.03% Triton and diluted to a concentration of 1–5 × 10^6^ spores/mL, which were than diluted 1,000 times in RPMI 1640 to achieve a 2-fold suspension more concentrated than the density needed or to approximately 2–4 × 10^3^ spores/mL (32). For filamentous fungi, conidia harvested from cultures grown for 3 days (Aspergillus spp.) or 5 days (*E.dermatitidis*) on SDA were suspended in sterile distilled water containing 0.03% Triton and diluted to a concentration of 2–5 × 10^6^ spores/mL, which were than diluted 100 times in RPMI 1640 to achieve a 2-fold suspension more concentrated than the density needed or to approximately 1–3 × 10^4^ spores/mL (33). The working concentration ranges of AZD8055, ITR, VRC, POS, and FLC were 1–64 μg/mL, 0.125–16 μg/mL, 0.125–16 μg/mL, 0.06–8 μg/mL, and 0.5–64 μg/mL against *Candida* spp. and 1–64 μg/mL, 0.06–8 μg/mL, 0.03–4 μg/mL,0.03–4 μg/mL, 0.5–64 μg/mL against C. neoformans complex, respectively. The working concentration ranges of AZD8055 and azoles (ITR, VRC and POS) against *E. dermatitidis* and azole-sensitive Aspergillus spp. were 1–64 μg/mL and 0.03–4 μg/mL, respectively. The working concentration ranges of AZD8055, ITR, VRC and POS against azole-resistant Aspergillus spp. were 1–64 μg/mL,0.25–32 μg/mL,0.25–32 μg/mL, and 0.03–4 μg/mL, respectively. As described, a 50 μl of AZD8055 with serial dilutions were inoculated in horizontal direction and another 50 μl of azoles with serial dilutions were inoculated in vertical direction on the 96-well plate, which contained 100 μl prepared inoculum suspension. Interpretation of results was performed after incubation at 35°C for 24h for *Candida* spp., 48h for C. neoformans complex, Aspergillus spp., and 72h for *E. dermatitidis*, respectively. The MICs applied for the evaluation of effects against *Candida* spp. and C. neoformans complex were determined as the lowest concentration resulting in 50% inhibition of growth (32). The MICs applied for the evaluation of effects against *E. dermatitidis* and Aspergillus spp. were determined as the lowest concentration resulting in 100% inhibition of growth (33). The combination interaction between AZD8055 and azoles was classified on the basis of the fractional inhibitory concentration index (FICI). The FICI as calculated by the formula: FICI=(Ac/Aa)+(Bc/Ba), where Ac and Bc are the MICs of antifungal drugs in combination, and Aa and Ba are the MICs of antifungal drugs A and B alone (35). An FICI of ≤0.5 is classified as synergy, an FICI of >0.5 to ≤4 indicates no interaction (indifference), and an FICI of >4 indicates antagonism (36). All tests were performed in triplicate.

### In *vivo* effect of AZD8055 alone and combined with azoles in Galleria mellonella.

Efficacy of AZD8055 alone and combined with azoles in G. mellonella infected with A. fumigatus *strain* AF002, *E. dermatitidis* strain BMU00038, C. auris strain 383, C. albicans strain R15, and C. neoformans complex strain Z2 were evaluated by survival assay as described previously (37), using sixth instar larvae (∼300mg, Sichuan, China). Groups of 20 larvae was maintained in wood shavings in the dark at room temperature before use. Suspensions of tested strains that had been grown on SDA for 72h at 37°C were harvested by gentle scraping of colony surfaces with sterile plastic loops, washed twice, and adjusted to 1 × 10^7^ spores/mL for *E. dermatitidis*, 1 × 10^8^ spores/mL for A. fumigatus, C. auris, C. albicans, and C. neoformans complex in sterile saline. The following control groups were included: larvae injected with 10 μl sterile saline, larvae injected with conidia suspension (5 μl for *E. dermatitidis*, 10 μl for A. fumigatus, C. auris, C. albicans, and C. neoformans complex), and untouched larvae. Conidia suspension and therapeutic and control solutions were injected into the larvae via the last right proleg using a Hamilton syringe (25 gauge, 50 μl). To determine the *in vivo* effects of AZD8055 alone and in combination with azoles against pathogenic fungi, a total of nine intervention therapy groups were included, AZD8055 treated group, ITC treated group, POS treated group, VRC treated group, FLC treated group (for C. albicans and C. neoformans complex only), AZD8055 with ITC treated group, AZD8055 with POS treated group, AZD8055 with VRC treated group and AZD8055 with FLC treated group (for C. albicans and C. neoformans complex only). Larvae were infected with conidia suspension and injected with tested agents (0.5 μg per agent) 2 h postinfection. The death of larvae was monitored by visual inspection of the color (brown-dark/brown) every 24 h for a duration of 5 days. The experiments were repeated triplicate using larvae from different batches. The G. mellonella survival curves were analyzed by the Kaplan–Meier method. Differences between groups were considered significant at *P* < 0.05.
