# Supplementary material for: In Vitro and In Vivo Interactions of TOR Inhibitor AZD8055 and Azoles against Pathogenic Fungi
Source: Microbiol Spectr. 2022 Jan 12;10(1):e02007-21. doi: 10.1128/spectrum.02007-21 (PMC8754115; doi:10.1128/spectrum.02007-21)
Supplement: SUPPLEMENTAL FILE 1 — Supplemental material. Download SPECTRUM02007-21_Supp_1_seq5.pdf, PDF file, 0.1 MB [file spectrum02007-21_supp_1_seq5.pdf]

Supplemental table 1 Source of tested strains

Strains	Isolation site	Underlying disease
Aspergillus spp.		
A. fumigatus		
AF293	Lung	Invasive aspergillosis
AF001	Lung	Invasive aspergillosis
AF002	Lung	Invasive aspergillosis
AF003	Lung	Invasive aspergillosis
AF004	Lung	Invasive aspergillosis
AF005	Lung	Invasive aspergillosis
AF006	Lung	Invasive aspergillosis
AF007	Lung	Invasive aspergillosis
AF008	Lung	Invasive aspergillosis
AF009	Lung	Invasive aspergillosis
AF010	Lung	Invasive aspergillosis
AF011	External auditory canal	Otomycosis
AF012	External auditory canal	Otomycosis
AF013	Lung	Invasive aspergillosis
R1(TR34/L98H)	Lung	Invasive aspergillosis
R2(TR34/L98H)	Lung	Invasive aspergillosis
R3(TR34/L98H)	Lung	Invasive aspergillosis
R4(TR46/Y121F/T 289A)	Lung	Invasive aspergillosis
A. flavus		
AFLA-1	Lung	Invasive aspergillosis
AFLA-2	Lung	Invasive aspergillosis
ALFA-3	Lung	Invasive aspergillosis
A. terreus		
AT1	Lung	Invasive aspergillosis
AT2	Lung	Invasive aspergillosis
Candida spp.		
C. auris		
381	External auditory canal	Otomycosis
382	Skin	Burn wound infection
383	Blood	Candidaemia
384	Blood	Candidaemia
385	Blood	Candidaemia
386	Blood	Candidaemia
387	Blood	Candidaemia
388	Blood	Candidaemia
389	BAL/lung	Pneumonia
390	Blood	Candidaemia
C. albicans		
R2	Vagina	Candidal vaginitis

R9	Vagina	Candidal vaginitis
R14	Oral cavit	Mycotic stomatitis
R15	Blood	Candidaemia
R65	Blood	Candidaemia
ATCC64550	Blood	Candidaemia
C. krusei		
ATCC00279	Blood	Candidaemia
C. parapsilosis		
ATCC22019	Blood	Candidaemia
C. glabrata		
BMU05448	Blood	Candidaemia
C. tropicalis		
BMU05150	Oral	Mycotic stomatitis
Cryptococcus neoformans complex		
Z1	Lung	Cryptococcal pneumonia
Z2	Lung	Cryptococcal pneumonia
Z3	Lung	Cryptococcal pneumonia
G5	Brain	Cryptococcal meningitis
G6	Brain	Cryptococcal meningitis
G7	Brain	Cryptococcal meningitis
G8	Brain	Cryptococcal meningitis
G9	Brain	Cryptococcal meningitis
G10	Brain	Cryptococcal meningitis
Exophiala dermatitidis		
BMU00028	Brain	Phaeohyphomycosis
BMU00029	Skin	Phaeohyphomycosis
BMU00030	Skin	Phaeohyphomycosis
BMU00031	Skin	Phaeohyphomycosis
BMU00034	Skin	Phaeohyphomycosis
BMU00035	Skin	Phaeohyphomycosis
BMU00036	Skin	Phaeohyphomycosis
BMU00037	Skin	Phaeohyphomycosis
BMU00038	Skin	Phaeohyphomycosis
BMU00039	Skin	Phaeohyphomycosis
BMU00041	Skin	Phaeohyphomycosis
109140	Skin	Phaeohyphomycosis
109144	Skin	Phaeohyphomycosis
109145	Skin	Phaeohyphomycosis
109148	Skin	Phaeohyphomycosis
109149	Skin	Phaeohyphomycosis
109152	Skin	Phaeohyphomycosis
